# Ileal Bile Acid Transporter Inhibition Reduces Post-Transplant Diarrhea and Growth Failure in FIC1 Disease—A Case Report

**DOI:** 10.3390/children9050669

**Published:** 2022-05-05

**Authors:** Johanna Ohlendorf, Imeke Goldschmidt, Norman Junge, Tobias Laue, Hamoud Nasser, Elmar Jäckel, Frauke Mutschler, Eva-Doreen Pfister, Diran Herebian, Verena Keitel, Ulrich Baumann

**Affiliations:** 1 Department of Kidney, Liver and Metabolic Diseases, Division of Pediatric Gastroenterology and Hepatology, Hannover Medical School, 30625 Hannover, Germany; goldschmidt.imeke@mh-hannover.de (I.G.); junge.norman@mh-hannover.de (N.J.); laue.tobias@mh-hannover.de (T.L.); nasser.hamoud@mh-hannover.de (H.N.); mutschler.frauke@mh-hannover.de (F.M.); pfister.eva-doreen@mh-hannover.de (E.-D.P.); baumann.u@mh-hannover.de (U.B.); 2 Department of Gastroenterology, Hepatology and Endocrinology, Hannover Medical School, 30625 Hannover, Germany; jaeckel.elmar@mh-hannover.de; 3 Department of General Pediatrics, Neonatology and Pediatric Cardiology, Medical Faculty, Heinrich-Heine University, 40225 Düsseldorf, Germany; herebian@med.uni-duesseldorf.de; 4 Department of Gastroenterology, Hepatology and Infectious Diseases, Otto-von-Guericke University, 39106 Magdeburg, Germany; verena.keitel-anselmino@med.ovgu.de; 5 Institute of Immunology and Immunotherapy, University of Birmingham, Birmingham B15 2TT, UK

**Keywords:** familial intrahepatic cholestasis 1 disease, *ATP8B1* deficiency, liver transplantation, children, diarrhea, IBAT inhibitor, Elobixibat, Odevixibat, case report

## Abstract

Familial intrahepatic cholestasis 1 (FIC1) disease is a genetic disorder characterized by hepatic and gastrointestinal disease due to *ATP8B1* deficiency, often requiring liver transplantation (LT). Extrahepatic symptoms, such as diarrhea, malabsorption, and failure to thrive, do not improve and instead may be aggravated after LT. We describe a patient with FIC1 disease who underwent LT at 2 years, 8 months of age. After LT, the child developed severe refractory diarrhea and failed to thrive. The response to bile acid resins was unsatisfactory, and the parents declined our recommendation for partial external biliary diversion (PEBD). Quality of life was extremely impaired, especially due to severe diarrhea, making school attendance impossible. Attempting to reduce the total bile acids, we initiated off-label use of the ileal bile acid transporter (IBAT) inhibitor Elobixibat (Goofice^™^), later converted to Odevixibat (Bylvay^™^). After six months of treatment, the patient showed less stool output, increased weight and height, and improved physical energy levels. The child could now pursue higher undergraduate education. In our patient with FIC1 disease, the use of IBAT inhibitors was effective in treating chronic diarrhea and failure to thrive. This approach is novel; further investigations are needed to clarify the exact mode of action in this condition.

## 1. Introduction

Familial intrahepatic cholestasis 1 (FIC1) disease is a genetic disorder caused by variants in ATPase Phospholipid Transporting 8B1 (*ATP8B1*), often requiring liver transplantation (LT) during childhood [1]. *ATP8B1* is expressed in various tissues, including the liver and intestines [2]. Extrahepatic symptoms, such as diarrhea and malabsorption, do not improve and are often aggravated after LT as a consequence of (presumed) chologenic diarrhea and impaired interaction between the transplanted liver and native bowel. *ATP8B1* deficiency reduces apical-membrane stability in various polarized cell types [3]. Due to normal liver *ATP8B1* function in the transplanted liver, the *ATP8B1*-deficient intestinal epithelium encounters a much higher chyme bile acid (BA) concentration than before LT. For healthy intestines, this is not a problem, but for intestines with FIC1 disease, their membrane vulnerability to BA results in chologenic diarrhea [4]. 

In 2021, the Kyoto group published data on 12 FIC1 disease patients, where 11 of the children suffered from chronic diarrhea after LT [5]. The second severe problem was liver steatosis and fibrosis, possibly caused by malabsorption, secondary to refractory diarrhea [6]. To date, medication such as bile acid resins seems to help in diarrhea, and especially surgical interventions, such as partial external biliary diversion (PEBD) after LT, can effectively alleviate diarrhea and can also reduce graft steatosis and inflammation [7,8,9]. Some centers perform a total internal biliary diversion at the time of LT [10]. 

We report on a patient with FIC1 disease who underwent LT at 2 years 8 months of age and developed severe refractory diarrhea early on. As the response to bile acid resins was unsatisfactory, and parents did not agree with an attempt at PEBD, we sought ethical approval for the off-label use of a high-dose ileal bile acid transporter inhibitor Elobixibat (Goofice^™^). After an encouraging clinical response, the child was converted to Odevixibat (Bylvay^™^) when compassionate use became available. 

Elobixibat, Odevixibat, or Maralixibat (Livmarli^™^), developed by different companies, are novel, orally available, selectively reversible IBAT inhibitors. Elobixibat is less potent than Odevixibat and is licensed as an anti-constipation treatment in Japan. They act locally in the gut via IBAT inhibition. First, they act in lowering BAs plasma or serum levels by the downregulation of BA reuptake in the distal ileum. Second, they act via improved colon clearance of BAs [11,12,13]. Odevixibat is approved for the treatment of progressive familial intrahepatic cholestasis in children older than 3 months [14]. Children with Alagille syndrome older than 1 year can be treated with Maralixibat in the case of cholestatic pruritus [13]. 

The enterohepatic circulation of BAs depends on the absorption of BA in the terminal ileum and colon. BAs play an essential role in the absorption and solubilization of cholesterol, dietary lipids, and fat-soluble vitamins [15]. Ninety-five percent of the BAs are reclaimed by the IBAT abundantly expressed in the terminal ileum [16]. IBAT inhibitors modulate enterohepatic BA circulation, enhancing the delivery of BAs to the colon, where they induce secretory and motor effects. Secondary effects of the inhibition of BA absorption are reduced activation of the farnesoid X receptor, decreased secretion of fibroblast growth factor-19 into the portal circulation, and increased BA synthesis [17]. During this process, the hepatic synthesis of BA is upregulated on account of the maintenance of enterohepatic circulation homeostasis [18]. Additionally, the abundant synthesis of cholesterol derivatives leads to the depletion of cholesterol stores in the liver, which simultaneously stimulates the expression of low-density lipoprotein (LDL) receptors on hepatocytes and reduces the level of serum LDL [19]. In trials, an increase in C4 (7α-hydroxy-4-cholesten-3-one) values were observed, which is an intermediate plasma marker in BA synthesis [20]. 

Another point is the influence of bile acids on the microbiome and their antibacterial effect. The molecular structures of primary BAs are modified by the gut bacteria and give rise to secondary BAs or other BA forms. Low levels of BAs in the gut appear to trigger an overgrowth of bacteria, with the consequence of inflammation and bacterial translocation [21]. The use of IBAT inhibitors changes the composition of the BA pool [22]. This can be seen in studies of BA composition in plasma and feces when using IBAT inhibitors. A substantial decrease in secondary and conjugate BAs was found [12]. 

Side effects reported in other studies are not serious. Maralixibat was well tolerated, and the most frequent adverse events were gastrointestinal [23]. The Elobixibat safety report on Japanese patients with chronic constipation (*n* = 979) showed that the proportion of adverse reactions was 12.4%, with diarrhea (2.9%), abdominal pain (1.4%), and constipation (0.8%) being the most frequent adverse events [24]. Odevixibat may decrease the adsorption of fat-soluble vitamins and may cause liver test abnormalities [14,25].

## 2. Case Report

We report on a male infant born by secondary cesarean section at term to healthy Caucasian and nonrelated parents with a birth weight of 3940 g. The pregnancy was uneventful, and there was no family history of liver disease. At three months of age, he developed gGT-negative cholestasis. FIC1 disease was genetically confirmed, revealing a homozygous nonsense variant (c.2788C>T, p.R930STOP) in *ATB8B1*. A liver biopsy performed at 4 months of age confirmed that the underlying diagnosis and fibrosis stage was ISHAK Fibrosis Score [26] with ISHAK F4. At this time, growth and development were normal on breastfeeding and formula milk; stool frequency was age-related with colored stools (laboratory values over time in Appendix A).

**Figure 1:**
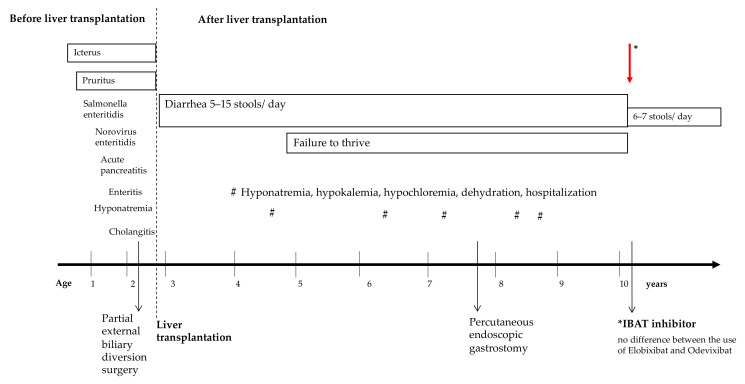
Life before and after liver transplantation over 10 years. IBAT, ileal bile acid transporter inhibitor.

**Figure 2:**
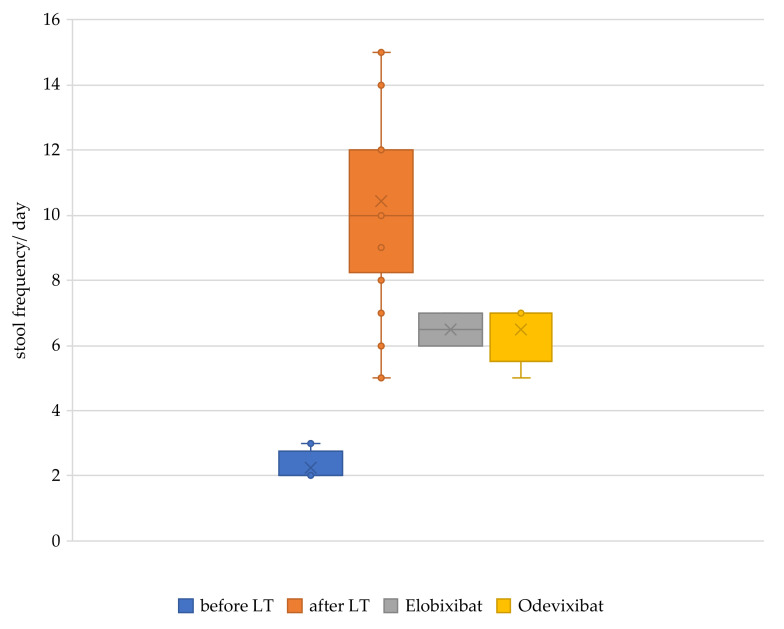
Daily stool frequency before liver transplantation, after liver transplantation, and with IBAT inhibitor. LT, liver transplantation; IBAT, ileal bile acid transporter inhibitor.

**Figure 3:**
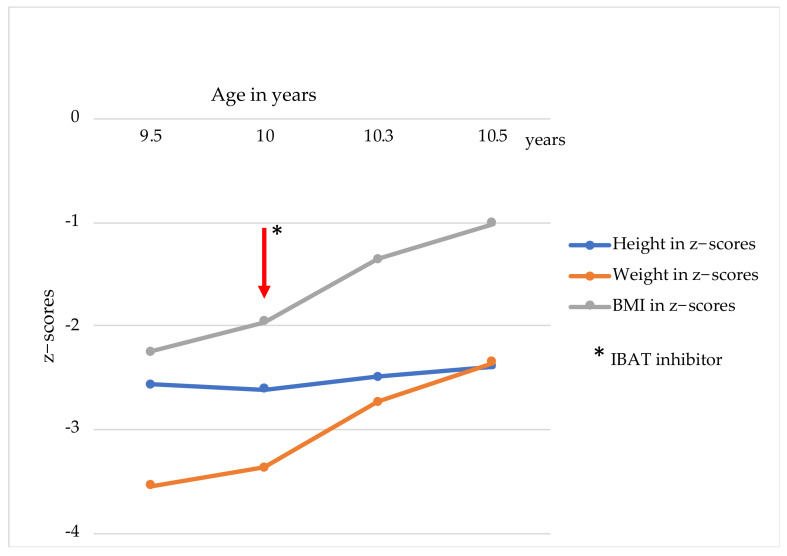
Weight, height, and BMI in z−scores before and with IBAT inhibitor. IBAT, ileal bile acid transporter inhibitor; BMI, body mass index.

At the age of 2 years and 1 month, the patient received partial external biliary diversion surgery (PEBD) because of persistent cholestasis and severe pruritus. In line with the previously performed liver biopsy, the fibrosis stage was still ISHAK F4. Following PEBD, postoperative complications arose, such as bleeding from the stoma with revision operation, cholangitis, high fluid loss with pronounced hyponatremia, and failure to thrive. As a further problem, signs of portal hypertension occurred due to progressive liver cirrhosis. A deceased donation of a full-size liver was performed at the age of 2 years and 8 months (Figure 1). Tacrolimus was chosen as a monotherapy immunosuppressant. Following LT, pruritus disappeared, and serum bilirubin concentration normalized. However, diarrhea progressively worsened within the first weeks following LT, with 10–13 liquid stools/day. Neither treatment with a bile acid sequestrant, an opioid-receptor agonist, an inhibitor of enkephalinase enzymes, or substitution of a pancreatic enzyme led to any significant change in stool consistency or frequency over the years after LT (Figure 2). The patients’ main problems were severe electrolyte derailments with dehydration and hospitalization in the 7 years after LT. Problems of hyponatremia, hypochloremia, and hypokalemia made a continuous oral substitution necessary. For this challenging management, the patient required a feeding tube (percutaneous endoscopic gastrostomy). Additionally, the patient suffered significantly from failure to thrive, beginning at the age of 5 years (Figure 3). 

At the age of 10 years, quality of life was extremely impaired, and the child was not able to attend normal school because of uncontrollable and frequent bowel movements. As the parents did not consent to repeat biliary diversion surgery to remedy chronic diarrhea, a therapy attempt with an IBAT inhibitor Elobixibat was made. After starting the treatment with 25 mg Elobixibat once a day, stool frequency decreased from a maximum of 15 stools/day to 6–7 stools/day. After one month, a change to Odevixibat on compassionate use became available. The initial dose of Odevixibat was 40 µg/kg/day and, while well-tolerated, was increased to 120 µg/kg/day once a day. In accordance with the safety profile, bilirubin, liver enzymes, and fat-soluble vitamins were monitored. There was no increase in liver enzymes or lack of fat-soluble vitamins during treatment (Table 1). There was no change in co-medication or substitution of electrolytes. A BA sequestrant was given in the same dosage all through this time. After 5 months of treatment, the family decided to change from a granule preparation of Tacrolimus to a prolonged-release preparation of the drug. The daily tacrolimus dosage whilst using IBAT inhibition was increased from 1.6 mg up to 6 mg to reach consistent therapeutic trough levels. 

The family noted a further improvement in stool frequency, appetite, and physical energy levels during the treatment with Elobixibat and later with Odevixibat. After seven months of treatment with an IBAT inhibitor, stool frequency has been stable at 6–7 times/day, making it possible for the child to partake in regular school lessons for the first time. Additionally, the patient is still showing improved weight development, with 24.5 kg (first percentile (P), −2.35 z)), height increase to 127 cm (first percentile (P), −2.39 z), and a significant change in BMI at 15.2 kg/m² (16P, −1.01 z) (Figure 3).

**Figure 4:**
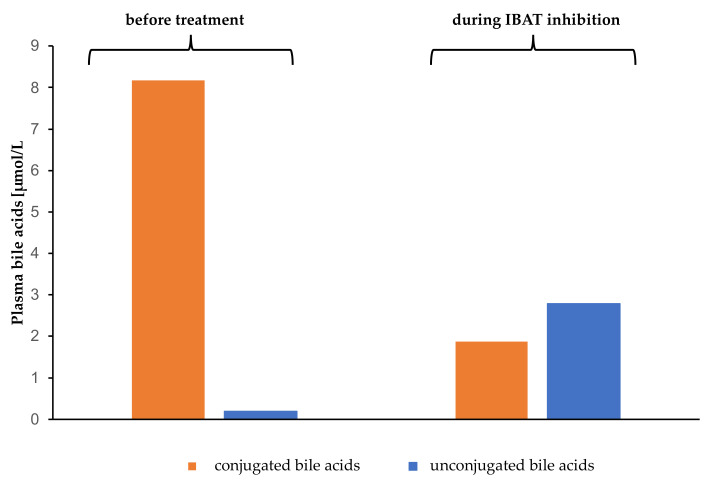
Conjugated and unconjugated plasma bile acids before and during treatment with IBAT inhibitors. IBAT, ileal bile acid transporter inhibitor.

**Figure 5:**
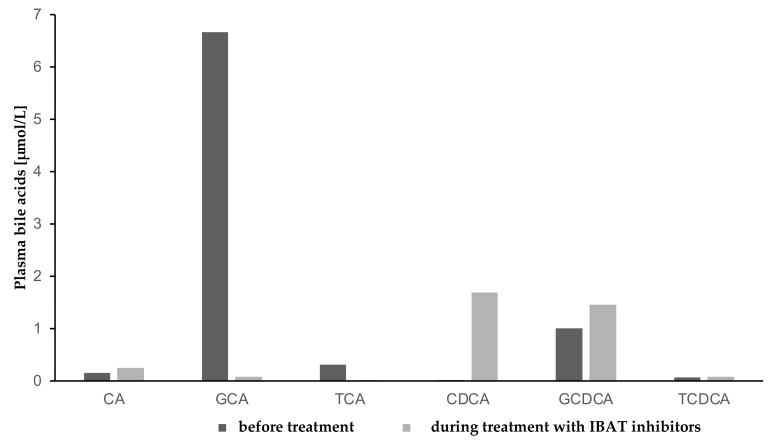
Primary bile acids and their conjugates in plasma before and during treatment with IBAT inhibitors. CA, Chenodeoxycholic acid; GCA, Glycocholic acid; TCA, Taurocholic acid; CDCA, Chenodeoxycholic acid; GCDCA, Glycochenodeoxycholic acid; TCDCA, Taurochenodeoxycholic acid, IBAT, ileal bile acid transporter inhibitor.

**Figure 6:**
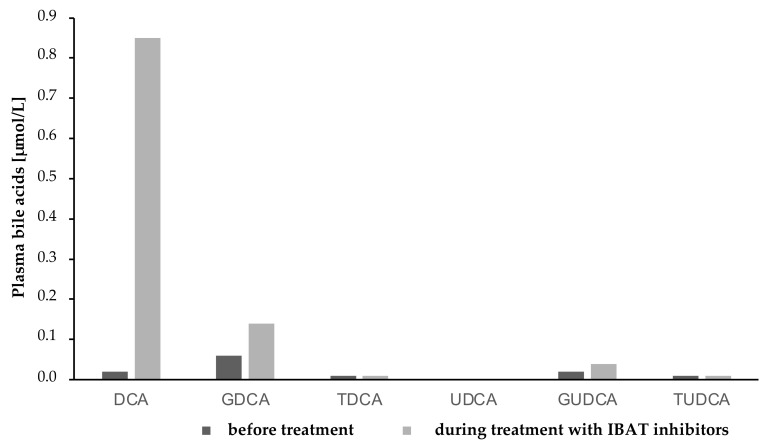
Secondary bile acids and their conjugates in plasma before and during treatment with IBAT inhibitors. DCA, Deoxycholic acid; GDCA, Glycodeoxycholic acid; TDCA, Taurodeoxycholic acid; UDCA, Ursodeoxycholic acid; GUDCA, Glycoursodeoxycholic acid; TUDCA, Tauroursodeoxycholic acid; IBAT, ileal bile acid transporter inhibitor.

Total plasma BAs were reduced by approximately 56% after 7 months of treatment with IBAT inhibitors. The composition of plasma BAs changed following Odevixibat administration, with a substantial decrease in taurocholic acid (TCA) and glycocholic acid (GCA), and an increase in levels of chenodeoxycholic acid (CDCA) and deoxycholic acid (DCA) (Figure 4, Figure 5 and Figure 6).

## 3. Discussion

We report on the successful medical treatment of a child with FIC1 disease suffering from debilitating diarrhea and failure to thrive post-liver transplantation. Intervention with IBAT inhibition rapidly stabilized the child and improved physical energy levels, appetite, and school performance. 

All currently available medication failed in our patient, and the parents refused surgical options. We therefore sought ethical approval to try a therapeutic attempt of IBAT inhibition. We began with Elobixibat as the only available and approved IBAT inhibitor at a dose 5 x times higher than the recommended starting dose in adults in order to mimic the effects that the IBAT inhibitors Odevixibat and Maralixibat have on the liver. In our patient, we saw a clear improvement in quality of life, primarily due to the decrease in diarrhea frequency and weight gain when taking IBAT inhibitors.

There are several possible explanations as to why diarrhea occurs after LT in *ATP8B1*-deficient patients. One is that direct BA toxicity is responsible, as both biliary diversion and BA binding resins seem to be effective treatments [27]. Partial biliary diversion diverts bile flow from the gut, thereby reducing high BA levels in chyme and most likely preventing high BA levels in the portal system and the liver.

Knisely et al. argue that *ATP8B1* deficiency disrupts farnesoid X receptor (FXR) synthesis and activation. In interaction with BA as a coligand, FXR normally regulates the synthesis of the apical sodium-dependent BA transporter (ASBT = IBAT, SLC10A2), which imports BA into the enterocytes. Furthermore, it regulates the synthesis of fibroblast growth factor 19 (FGF19), which suppresses the synthesis of hepatic BAs. *ATP8B1*-diseased bowel and liver may be primarily FXR-deficient or unable to activate FXR. Accordingly, this leads to LT and the fact that when physiological levels of BA challenge the intestine, inappropriately low levels of FGF19 and inappropriately high levels of BA (via unregulated ASBT) are released into the portal venous blood. As a result, the liver can secrete large amounts of both recirculated and newly synthesized BA, adding even more BA to the enterohepatic circulation [4]. 

Graffner et al. showed a substantial decrease in total BA in plasma when using IBAT inhibitors in healthy volunteers [12]. We saw the same effect in our patient, with decreasing total BA in plasma during treatment with Odevixibat and a significant change of BA composition in plasma when using IBAT inhibitors. In contrast to Graffner’s results, our patient showed a substantial decrease in primary GCA and TCA and an increase in the primary, unconjugated BA CDCA and secondary BA DCA. It must be taken into account that our patient, with underlying *ATP8B1* deficiency, was treated with IBAT inhibitors after LT, and the cohort which Graffner examined were healthy adults. We assume a residual function of the ileal bile acid receptor in our patient, which could have been blocked by the medication.

Partial biliary diversion in *ATP8B1*-deficient patients who undergo LT seems efficacious in both diarrhea and allograft steatosis, as it diverts bile flow from the gut, thereby reducing high BA levels in chyme and, most likely, preventing high BA levels in the portal system and the liver. As a nonsurgical approach, IBAT inhibitors are an alternative way of disrupting enterohepatic BA circulation. They work by inhibiting the IBAT that reabsorbs BA from the intestines. IBAT inhibition has been shown to reduce serum bile acids and pruritus in trials in pediatric cholestatic liver diseases [28]. Effects of Odevixibat and Maralixibat on BA in cholestatic Alagille Syndrome patients have been thoroughly demonstrated [29,30]. Karpen and colleagues discussed the novel potential of IBAT inhibitors such as Odevixibat or Maralixibat for use in children with biliary atresia as a means to reduce hepatic bile acid concentration after Kasai portoenterostomy. By reducing the return of bile acids to the cholestatic liver, IBAT inhibitors may potentially lessen or delay liver damage associated with the hepatotoxicity and cholangiopathy of bile acid accumulation [31]. In 2020, Slatventinsky and Sturm described a patient with PFIC type 2 and severe pruritus. Both treatment with Odevixibat and PEBD resulted in a similar normalization of serum BA levels as well as a reduction in pruritus and improvement in sleep quality. Therefore, Odevixibat may be potentially equivalent to surgical PEBD in reducing serum BAs and cholestatic pruritus [32]. We therefore assume that the same effect that leads to a reduction in the bile acid pool due to PEBD is present in IBAT-inhibitor-treated patients and, thus, has an effect on chologenic diarrhea, as seen in our patient.

## 4. Conclusions

To date, partial biliary diversion in patients with FIC1 disease who undergo LT appears to be efficacious in treating both diarrhea and allograft steatosis. In our patient, a similar clinical response could be observed with the use of different IBAT inhibitors. This approach is novel, and it proved effective in terms of clinical outcomes, such as decreased daily stool frequency, weight and appetite gain, increased physical energy levels, and overall quality of life.

## Figures and Tables

**Table 1:** Liver enzymes, fat-soluble vitamins before and during treatment. IBAT, ileal bile acid transporter inhibitor; ALAT, alanine aminotransferase; ASAT, aspartate aminotransferase; INR, international normalized ratio.

Laboratory Parameters	Before Treatment	After 3 Months IBAT Inhibitor	After 7 Months IBAT Inhibitor	Normal Range
Bilirubin (µmol/L)	3	3	3	3–17
ALAT (U/I)	33	42	37	<35
ASAT (U/I)	29	50	45	<45
INR	1.01	0.97	0.98	0.90–1.25
Vitamin A (mg/L)	0.21	0.36	0.43	0.3–0.06
Vitamin E (mg/L)	10.3	11.67	13.82	3–9
25-OH-D3 (ng/mL)	32.8	40.3	45	20–70
Tacrolimus (µg/L)	3.3	1.5	1.8	2–5

## Data Availability

Data are available from the corresponding author on request.

## Appendix A

**Table A1:** Liver enzymes, fat-soluble vitamins (under substitution) at the age of 1 year, before PEBD and before LT. PEBD, partial external biliary diversion; LT, liver transplantation; ALAT, alanine aminotransferase; ASAT, aspartate aminotransferase; INR, international normalized ratio.

Laboratory Parameters	Age of 1 Year	Before PEBD	Before LT	Normal Range
Bilirubin (µmol/L)	71	170	70	3–17
ALAT (U/I)	39	72	56	<35
ASAT (U/I)	94	147	113	<45
INR	1.11	1.20	1.25	0.90–1.25
Vitamin A (mg/L)	0.39	0.17	0.33	0.3–0.06
Vitamin E (mg/L)	13.34	7.36	5.65	3–9
25-OH-D3 (ng/mL)	n.d.	13.3	32.0	20–70
